# Preferential looking studies of trustworthiness detection confound structural and expressive cues to facial trustworthiness

**DOI:** 10.1038/s41598-022-21586-6

**Published:** 2022-10-21

**Authors:** Adam Eggleston, Maria Tsantani, Harriet Over, Richard Cook

**Affiliations:** 1 Department of Psychology, University of York, York, YO10 5DD UK; 2 Department of Psychological Sciences, Birkbeck, University of London, London, UK

**Keywords:** Psychology, Human behaviour

## Abstract

On encountering a stranger, we spontaneously attribute to them character traits (e.g., trustworthiness, intelligence) based on their facial appearance. Participants can base impressions on structural face cues—the stable aspects of facial appearance that support identity recognition–or expression cues, such as the presence of a smile. It has been reported that 6- to 8-month-old infants attend to faces that adults judge to be trustworthy in preference to faces judged untrustworthy. These results are striking because the face stimuli employed were ostensibly emotion neutral. Consequently, these preferential looking effects have been taken as evidence for innate sensitivity to structural face cues to trustworthiness. However, scrutiny of the emotion rating procedure used with adults suggests that the face stimuli employed may have been judged emotion neutral only when interleaved with more obvious examples of facial affect. This means that the faces may vary in emotional expression when compared to each other. Here, we report new evidence obtained from adult raters that the stimuli used in these studies confound trustworthiness and untrustworthiness with the presence of happiness and anger, respectively. These findings suggest that the preferential looking effects described in infants are compatible with a preference for positive facial affect and may not reflect early sensitivity to structural face cues to trustworthiness.

## Introduction

When we first encounter a stranger, we spontaneously attribute to them a wide variety of character traits based on their facial appearance; for example, inferring their apparent trustworthiness, competence and intelligence^1,2^. Despite the fact that they have little or no basis in reality, these first impressions exert a strong influence on our behaviour^3^. First impressions from facial appearance have been shown to affect financial decisions^4^, legal judgements and criminal sentencing decisions^5^, and voting patterns in elections^6,7^.

When asked to evaluate the traits of people depicted in stimulus images, participants can base their judgements on different cues. One source of information is facial structure; i.e., permanent or semi-permanent aspects of facial appearance. These are the same cues that support judgements of facial identity and include feature shape and configuration^8–10^. First impressions based on facial structure include the inference of trustworthiness from facial width-to-height ratio^11^, babyfacedness^12,13^, sexually dimorphic cues^14^, and perceived ethnicity^15^. A second source of information on which participants can base trait judgements is facial expression. For example, smiling faces are more likely to be judged trustworthy, while angry faces are more likely to be judged untrustworthy^16–19^. Neuroscientific and neuropsychological data converge on the view that perceptual sensitivity to facial structure and facial expression dissociates^8–10,20^.

### The origin and development of first impressions

There is growing interest in the developmental trajectory of first impressions. To date, most developmental studies have focussed on attributions of trustworthiness^17,21–24^. A recent systematic review and meta-analysis found that reliable judgements of trustworthiness emerge around 3 − 5 years of age, and that trust impressions continue to develop throughout childhood, showing adult-like patterns between 10 and 13 years of age^25^. This conclusion accords well the view that first impressions are learned ontogenetically, either through first-hand interaction with others, or through exposure to cultural messages about the appearance of heroes and villains, ‘jocks and ‘geeks’, the competent and incompetent^26–29^.

Nevertheless, certain results support nativist accounts of first impressions that posit some form of innate face-trait knowledge^30,31^. In particular, Jessen and Grossmann^23^ reported that 7-month-old infants attended to faces that adults judged to be trustworthy in preference to faces that adults judged as neutral or untrustworthy. In a follow-up study, Sakuta and colleagues^24^ found that 6–8 month-old infants preferentially attended to trustworthy faces relative to untrustworthy faces—replicating the results of Jessen and Grossmann^23^–but only when faces were high in dominance. There was no effect of trustworthiness when faces were submissive (i.e., low in dominance). These data appear incompatible with a learning account of first impressions^26,28^.

These results are striking because they were obtained with stimuli that were ostensibly “emotion neutral”. It is known that young infants show some crude recognition and understanding of facial emotion^32–34^. Hence, evidence that 6–8-month-old infants attend preferentially to positive facial affect would not be particularly surprising. In the absence of expression cues, however, these results have been taken as evidence that 6–8-month-old infants exhibit early sensitivity to structural face cues to trustworthiness. For example, Jessen and Grossmann^35^ assert: “*Infants at the age of 7 months have been shown to detect changes in facial trustworthiness and preferentially look at trustworthy faces when presented supraliminally (…). While it is unlikely that infants possess an elaborate concept of trustworthiness, they do differentiate between trustworthy and untrustworthy faces based on subtly different featural combinations… In this context, it is important to consider that facial trustworthiness detection is based on invariant (stable) facial information rather than the variant (transient) facial information*” (p457).

### The present study

The stimulus images used by Jessen and Grossmann^23^ and Sakuta and colleagues^24^ were taken from a collection of synthetic faces created by Oosterhof and Todorov^19^ using FaceGen Modeller 3.2 (Singular Inversions, 2007, Toronto, Canada). Oosterhof and Todorov^19^ applied parametric manipulations to different source models to produce face images that varied in their apparent trustworthiness and / or dominance. With respect to facial emotion, Jessen and Grossmann^23^ explain: “*…although faces in which trustworthiness or untrustworthiness is extremely exaggerated (beyond* ± *3 SD) have been shown to be perceived as happy or angry by adults (…), the facial stimuli used in the current study were within this critical* ± *3 SD range and are thus still perceived as emotionally neutral by adult raters (…).*”

Importantly, however, the emotion rating data that Jessen and Grossmann cite were collected using a procedure that may have been insensitive to subtle facial emotions^19^. Specifically, the faces used by Jessen and Grossmann^23^ and Sakuta and colleagues^24^ were interleaved with faces that contained more salient cues to facial emotion when they were judged to be emotion neutral by adult raters. The presence of more obvious examples of facial affect may have altered the decision criteria applied by participants when judging the kinds of image used by Jessen and Grossmann^23^ and Sakuta and colleagues^24^. Crucially, the trustworthy and untrustworthy faces used in these studies may not appear emotion neutral when compared to each other. By way of analogy, an accountant and a librarian may be judged to have a relatively typical standard of living compared to a billionaire. However, when compared to each other, the accountant may be judged relatively wealthy.

If the trustworthy and untrustworthy stimuli employed by Jessen and Grossmann^23^ and Sakuta and colleagues^24^ were found to differ systematically in terms of facial emotion, this would raise the possibility that the preferential looking effect may be driven by a simple preference for positive facial affect rather than innate knowledge about the invariant face structure of trustworthy people. We investigated this possibility in two experiments in which we subjected the stimuli used by Jessen and Grossmann (Experiment 1) and Sakuta and colleagues (Experiment 2) to a more sensitive emotion rating procedure^36^. Adult participants evaluated the emotional content of the stimuli used in the two studies and only these stimuli. This meant that the decision criteria applied were not distorted by the presence of irrelevant images. We elected to focus on two emotions in particular—happiness and anger–because the presence of these emotions is known to strongly influence impressions of trustworthiness^16–19^.

## Experiment 1

In our first experiment, we considered the stimuli employed by Jessen and Grossmann^23^. We hypothesised that the trustworthy faces used by Jessen and Grossmann^23^ would be rated higher than the neutral and the untrustworthy faces on a measure of happiness and that the untrustworthy faces would be rated higher than the trustworthy and the neutral faces on a measure of anger. The sample-size, inclusion criteria, study design and the intended analyses were pre-registered. This information and corresponding data can be found on the OSF (https://osf.io/dpxgw/?view_only=dfca636b4b2741f8882476b58afced13).

### Method

#### Participants

100 adult participants (*M*_age_ = 35.17, *SD*_age_ = 12.44; 63 female, 35 male, 2 non-binary) were recruited via Prolific (www.prolific.co). All participants were fluent in English and reported that their current country of residence was the U.K. No-one was replaced or excluded. Power analysis conducted with G-Power 3.1 indicated that a sample of 97 ensured a paired-samples *t*-test had 90% power to detect an effect size of 0.30. This was rounded up to 100.

#### Stimuli and procedure

**Figure 1:**
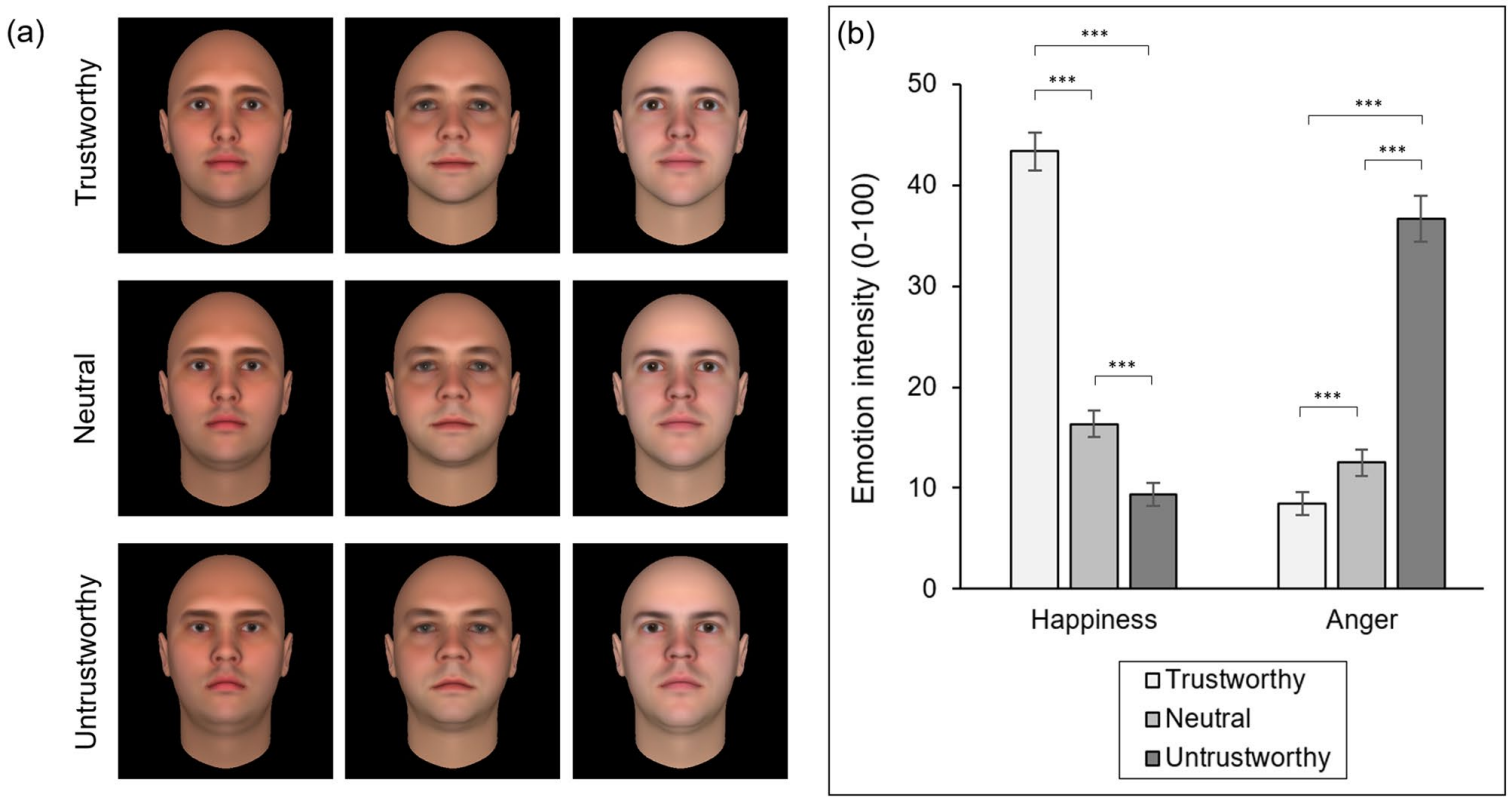
Stimuli and results for Experiment 1. (**a**) The nine stimulus images employed by Jessen and Grossmann^23^. (**b**) Mean emotion intensity ratings for the three types of face. Error bars denote ± SEM. *** denotes *p* < 0.001.

The nine face stimuli (see Fig. 1a) were the same nine images used by Jessen and Grossmann^23^. These faces were sourced from the collection created by Oosterhof and Todorov^19^. The nine images were derived from three source identities. From each identity, three faces were derived that varied systematically in apparent trustworthiness (untrustworthy, neutral, trustworthy). The apparent dominance of these faces was not manipulated.

Participants rated the nine stimuli one at a time, in a randomised order. Following a fixation cross (1000 ms), each face was presented at the centre of the display (3000 ms). Participants were then asked to rate how happy and angry each face appeared using two scales ranging from 0 (Not at all) to 100 (Extremely). Participants were specifically instructed that if they thought a face showed no signs of happiness or anger, they should set both sliders to zero. For the purpose of the analysis described below, we averaged the ratings of happiness and anger awarded to the three exemplars of each face type (neutral, trustworthy, untrustworthy). Both of the experiments described were conducted online via Gorilla Experiment Builder (https://gorilla.sc/).

#### Statistical procedures

In both studies, participants’ emotion ratings were evaluated using repeated measures ANOVA and paired-samples *t*-tests (α = 0.05, two-tailed), performed using SPSS v.28. Where sphericity could not be assumed, the Greenhouse–Geisser correction was applied. For the ANOVAs, we report partial eta squared (η^2^_p_) as a measure of effect size. For the paired *t*-tests, we report Cohen’s *d*, calculated by dividing the mean pairwise difference by the standard deviation of the pairwise differences.

### Results

The mean ratings were subjected to ANOVA with Trustworthiness (untrustworthy, neutral, trustworthy) and Emotion (happiness, anger) as within-subjects factors (see Fig. 1b). The analysis revealed a significant main effect of Trustworthiness [*F* (1.79, 177.64) = 130.49, *p* < 0.001, η^2^_p_ = 0.57] whereby emotion ratings were generally lower for neutral faces than for trustworthy or untrustworthy faces, and a significant main effect of Emotion [*F* (1, 99) = 12.38, *p* < 0.001, η^2^_p_ = 0.11] whereby happiness ratings were generally higher than anger ratings. In line with our pre-registered predictions, we also observed a significant Trustworthiness × Emotion interaction [*F* (1.26, 124.27) = 353.25, *p* < 0.001, η^2^_p_ = 0.78].

Happiness ratings were highest for trustworthy faces (*M* = 43.37, *SD* = 18.88), followed by neutral faces (*M* = 16.37, *SD* = 13.25), and lowest for untrustworthy faces (*M* = 9.42, *SD* = 11.37). Happiness ratings awarded to trustworthy faces exceeded those awarded to neutral faces [*t* (99) = 22.26, *p* < 0.001, *d* = 2.23] and untrustworthy faces [*t*(99) = 20.66, *p* < 0.001, *d* = 2.07]. The happiness ratings awarded to the neutral faces also exceeded those given to the untrustworthy faces [*t* (99) = 7.52, *p* < 0.001, *d* = 0.75].

Anger ratings were highest for untrustworthy faces (*M* = 36.71, *SD* = 22.29), followed by neutral faces (*M* = 12.50, *SD* = 13.11), and lowest for trustworthy faces (*M* = 8.47, *SD* = 11.60). Anger ratings awarded to untrustworthy faces exceeded those awarded to neutral faces [*t* (99) = 15.825, *p* < 0.001, *d* = 1.58] and trustworthy faces [*t* (99) = 15.05, *p* < 0.001, *d* = 1.51]. The anger ratings awarded to the neutral faces also exceeded those given to the trustworthy faces [*t* (99) = 5.15, *p* < 0.001, *d* = 0.52].

These results demonstrate that, when rated with an appropriately sensitive procedure, the trustworthy and untrustworthy facial stimuli used by Jessen and Grossmann^23^ *do* vary systematically in their emotional expressions. In light of these data, the conclusion of Jessen and Grossmann that their preferential looking effect reflects sensitivity to structural cues to trustworthiness appears premature. In Experiment 2, we assess whether the trustworthy and untrustworthy stimuli used by Sakuta and colleagues^24^ also vary systematically in their facial emotion content.

## Experiment 2

Since the publication of Jessen and Grossmann’s preferential looking result^23^, Sakuta and colleagues^24^ published a partial replication of their findings. They found that 6–8-month-old infants preferentially attended to trustworthy faces relative to untrustworthy faces, but only when faces were also manipulated to appear dominant–there was no effect of facial trustworthiness on preferential looking when the target faces were manipulated to appear submissive. The results of our first experiment suggest that the preferential looking effect may be driven by the presence of facial emotion rather than structural cues to facial trustworthiness. In our second experiment we investigated whether differences in facial emotion present in the authors’ four stimulus images may explain the pattern of results described by Sakuta and colleagues^24^. We predicted that their trustworthy faces would be rated as happier than their untrustworthy faces, and that their untrustworthy faces would be rated as angrier than their trustworthy faces. However, we hypothesized that these differences may be greater for the dominant faces than for the submissive faces. Once again, our sample-size, inclusion criteria, study design and intended analysis were pre-registered. This information and corresponding data can be found on the OSF (https://osf.io/dpxgw/?view_only=dfca636b4b2741f8882476b58afced13).

### Method

#### Participants

A further 100 adult participants (*M*_age_ = 37.74, *SD*_age_ = 12.70; 74 female, 23 male, 3 non-binary) were recruited via Prolific (www.prolific.co). Once again, all participants were fluent in English and reported that their current country of residence was the U.K. No-one was replaced or excluded. None of the participants from Experiment 1 took part in Experiment 2.

#### Stimuli and procedure

**Figure 2:**
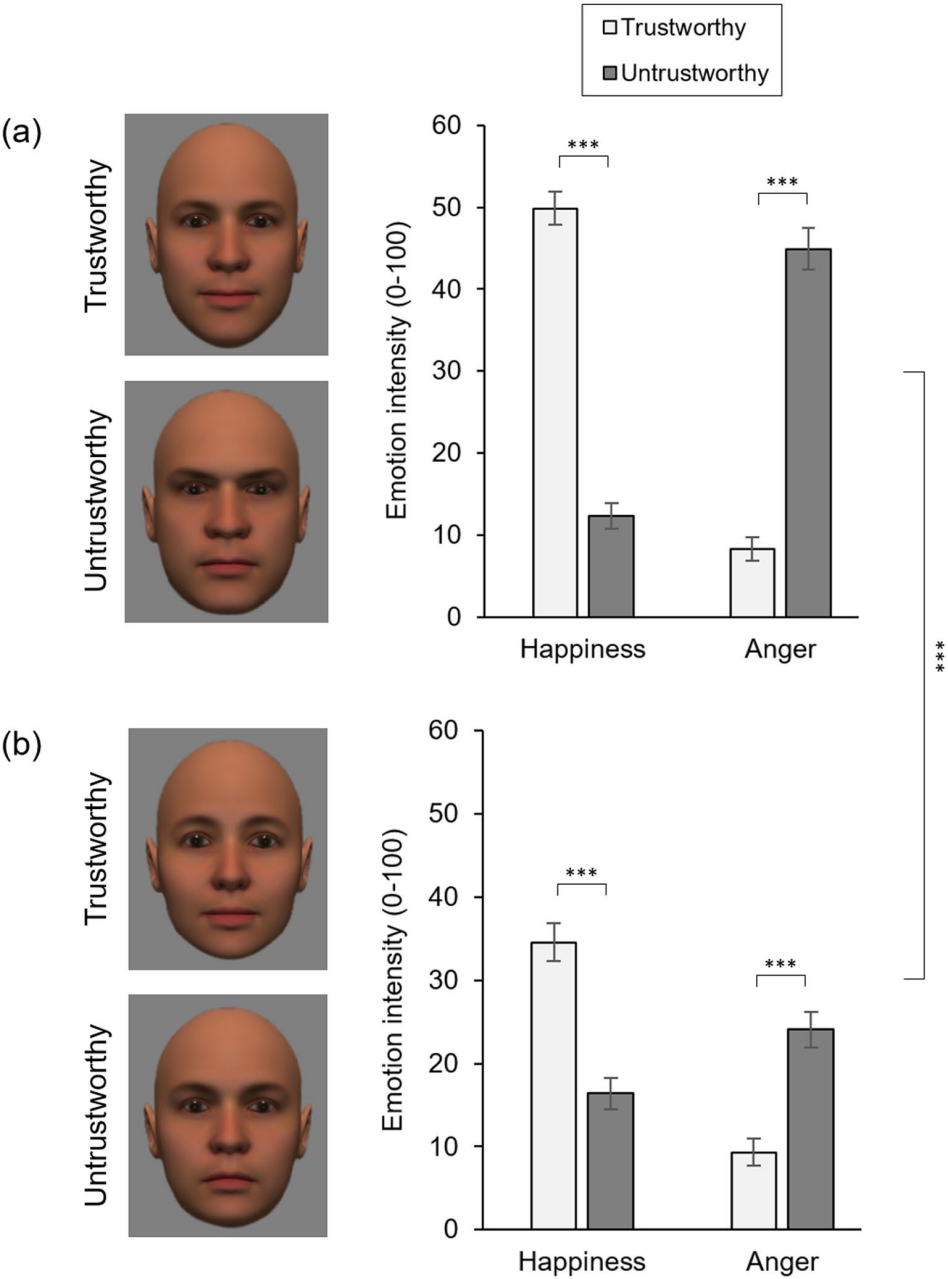
Stimuli and results for Experiment 2. (**a**) The dominant face stimuli used by Sakuta and colleagues^24^ (left) and the associated emotion ratings (right). (**b**) The submissive face stimuli used by Sakuta and colleagues^24^ (left) and the associated emotion intensity ratings (right). Error bars denote ± SEM. *** denotes *p* < 0.001.

The four face stimuli used in Experiment 2 (see Fig. 2a) were the same four images used by Sakuta and colleagues^24^. Once again, these stimuli were sourced from the set created by Oosterhof and Todorov^19^. The four images used were created from a single source identity by simultaneously applying manipulations of trustworthiness and dominance. The resulting images comprised a trustworthy-dominant variant, a trustworthy-submissive variant, an untrustworthy-dominant variant, and an untrustworthy-submissive variant. With the exception of the stimuli used, the rating procedure was identical to that described in Experiment 1.

### Results

The ratings were subjected to ANOVA with Trustworthiness (untrustworthy, trustworthy), Dominance (dominant, submissive) and Emotion (happiness, anger) as within-subjects factors (see Fig. 2a). The analysis revealed a significant main effect of Dominance [*F* (1, 99) = 65.89, *p* < 0.001, η^2^_p_ = 0.40] whereby emotion ratings were generally lower for submissive faces than for dominant faces, and a significant main effect of Emotion [*F* (1, 99) = 13.94, *p* < 0.001, η^2^_p_ = 0.12] whereby happiness ratings were generally higher than the anger ratings. Neither the Trustworthiness × Dominance interaction [*F* (1, 99) = 0.54, *p* = 0.463, η^2^_p_ = 0.01], nor the Dominance × Emotion interaction [*F* (1, 99) = 3.78, *p* = 0.055, η^2^_p_ = 0.04], reached significance.

As expected, we observed a significant Trustworthiness × Emotion interaction [*F* (1, 99) = 487.03, *p* < 0.001, η^2^_p_ = 0.83]. Higher levels of happiness were seen in the trustworthy faces than in the untrustworthy faces, in both the dominant (*M*_trust_ = 49.87, *SD*_trust_ = 20.31; *M*_untrust_ = 12.35, *SD*_untrust_ = 15.77) [*t* (99) = 18.36, *p* < 0.001, *d* = 1.84] and submissive (see Fig. 2b; *M*_trust_ = 34.57, *SD*_trust_ = 22.36; *M*_untrust_ = 16.43, *SD*_untrust_ = 18.93) [*t* (99) = 8.69, *p* < 0.001, *d* = 0.87] variants. Similarly, higher levels of anger were seen in the untrustworthy faces than in the trustworthy faces, in both the dominant (*M*_untrust_ = 44.92, *SD*_untrust_ = 25.51; *M*_trust_ = 8.25, *SD*_trust_ = 14.29) [*t* (99) = 15.38, *p* < 0.001, *d* = 1.54] and submissive (*M*_untrust_ = 24.09, *SD*_untrust_ = 21.55; *M*_trust_ = 9.29, *SD*_trust_ = 16.24) [*t* (99) = 7.55, *p* < 0.001, *d* = 0.76] variants.

Importantly, however, the Trustworthiness × Emotion interaction varied as a function of Dominance [*F*(1, 99) = 80.99, *p* < 0.001, η^2^_p_ = 0.45]. In order to understand this interaction, we computed for each participant ΔHappiness (the happiness rating awarded to the trustworthy face *less* the happiness rating awarded to the untrustworthy face) and ΔAnger (the anger rating awarded to the untrustworthy face *less* the anger rating awarded to the trustworthy face) for the dominant and submissive variants. Paired *t*-tests revealed that ΔHappiness [*t*(99) = 6.85, *p* < 0.001, *d* = 0.69] and ΔAnger [*t*(99) = 7.64, *p* < 0.001, *d* = 0.76] were both greater for the dominant faces, than for the submissive faces.

### Ethical approval

This study was approved by the University of York Department of Psychology’s Ethics Committee (approval #798). All methods were performed in accordance with the committee’s guidelines and performed in accordance with the Declaration of Helsinki.

### Informed consent

In both experiments reported in the manuscript, informed consent was obtained from all participants.

## Discussion

Jessen and Grossmann^23^ reported that 7–month-old infants preferentially attended to faces that adults judged to be trustworthy over faces that adults judged to be trust neutral or untrustworthy. According to Jessen and Grossmann^23^, this effect could not be explained by a preference for positive facial affect because adult raters had previously judged the nine stimulus images to be emotion neutral^19^. As such, they argue that the preferential looking observed reflects early sensitivity to structural cues to facial trustworthiness^23,35,37^. This would be a striking finding, potentially suggestive of innate face-trait knowledge.

However, the emotion rating data cited by Jessen and Grossmann were obtained using a procedure that was likely to be insensitive to subtle emotion cues^19^. The images used by Jessen and Grossmann^23^ may have been judged “emotion neutral” only when compared to the more obvious examples of facial affect with which they were interleaved. In our first experiment, we asked adults to rate the emotional expressions of the faces used by Jessen and Grossmann^23^ in the absence of any other images. We found clear evidence that Jessen and Grossmann’s manipulation of facial trustworthiness was confounded with the presence of facial emotion. The trustworthy stimuli were judged to be happier than the neutral and untrustworthy faces, while the untrustworthy stimuli contained more anger than the neutral and trustworthy faces.

In our second experiment, we examined the stimuli used by Sakuta and colleagues^24^ using the same procedure. In this study, the authors were able to replicate the preferential looking effect described by Jessen and Grossmann^23^ in 6–8 month-old infants with trustworthy and untrustworthy faces that were dominant, but not with trustworthy and untrustworthy faces that were submissive. Overall, we found that the trustworthy faces used by Sakuta and colleagues^24^ were judged to be happier and less angry than the untrustworthy faces. Crucially, however, the strength of the emotion confound was stronger for the dominant faces (the pair that produced the preferential looking effect) than for the submissive faces (the pair that failed to produce the preferential looking effect). Together, these findings suggest that the preferential looking effects described by Jessen and Grossmann^23^ and Sakuta and colleagues^24^ may well reflect early sensitivity to facial emotion (e.g., a preference for positive affect), not early sensitivity to structural cues to facial trustworthiness.

Some people may have a facial structure (e.g., narrow eyes; a mouth that naturally curves upwards at the corners) that means that observers perceive emotion where none is experienced or conveyed. Consequently, one could argue that the stimuli used by Jessen and Grossmann^23^ and Sakuta and colleagues^24^ should be considered ambiguous; they could be perceived as people with unusual face shapes expressing no emotion, or as people with more typical face shapes expressing subtle signs of happiness and anger^19,38^. Crucially, however, perception is probabilistic and inferential^39–41^. The present data confirm that when confronted with these images, adult observers perceive people with statistically likely face shapes expressing emotion, rather than people with statistically unlikely face shapes expressing no emotion. When addressing questions of mechanism and origin–how and why we spontaneously infer the traits of others–it makes little difference whether traits are inferred from veridical expression cues (where the person depicted experiences or intends to convey an emotion) or pseudo-expression cues (where the observer perceives emotion where none is experienced or conveyed). In both cases, the means by which participants infer traits is likely to be the same^26^.

### Limitations and directions for future research

Previous reports that infants prefer to look at trustworthy faces over untrustworthy faces have been taken as evidence that they possess innate knowledge about the facial structure of trustworthy individuals. Our results (obtained with adult participants) suggest a different possibility: that these preferential looking results may simply be attributable to the different expression cues present in the trustworthy and untrustworthy facial stimuli used in these studies. At present, however, that is all our results do–suggest a different type of explanation. We cannot say for sure which type of cue, structural or expression, is responsible for infants’ preferential looking behaviour. A definitive answer to this question will require data from infant participants.

One way to address this question would be to examine infants’ fixation behaviour using facial stimuli that vary in trustworthiness, but that are closely matched in terms of their expressions. If infants prefer to look at apparently trustworthy faces over apparently untrustworthy faces, the effect should still be seen using this approach. However, if the preferential looking described by Jessen and Grossmann^23^ is attributable to differences in facial expression, no systematic preference should be seen. The results from the low-dominance condition of Sakuta and colleagues^24^ provide some early indication of the latter.

Jessen and Grossmann^23^ and Sakuta and colleagues^24^ used stimuli from the database generated by Oosterhof and Todorov^19^ in the belief that trustworthiness manipulations of three standard deviations or less do not influence how adults perceive the model’s facial emotion. Our results suggest that this assumption is unsafe, at least for the models examined in the present study. Future research may seek to examine how widespread this problem is; for example, whether it is true of other models generated by Oosterhof and Todorov^19^. A great many studies in the first impressions literature have used stimuli from this collection–including work investigating the neural underpinnings^37,42,43^ and behavioural consequences^44–46^ of first impressions, and comparative research^47^–presuming that stimuli within the ± 3 SD range are perceived as emotion-neutral by human adults. In some cases, findings attributed to differences in facial structure may actually reflect perceived differences in facial expression.

In the present study, we focussed on the presence of two emotions, happiness and anger, that are known to affect judgements of facial trustworthiness. By restricting our examination to just two emotions, we sought to avoid statistical problems arising from numerous pairwise comparisons. Nevertheless, it is possible that the trustworthy and untrustworthy stimuli considered differ systematically in other emotions. For example, one might expect to see similar results for anger and disgust, which are often confused^36,48^ and are located close to one another in Russell’s circumplex space^49^ (i.e., they are both high-arousal and associated with negative valance).

## Conclusion

Previous reports suggest that 6- to 8-month-old infants attend to faces that adults judge to be trustworthy in preference to faces adults judge to be untrustworthy^23,24^. Because the face stimuli used in these studies were purportedly emotion-neutral, these preferential looking effects have been taken as evidence for innate sensitivity to structural face cues to trustworthiness^35,50^. However, the findings described here indicate that the stimuli used in these studies were not emotion-neutral. Rather, the trustworthy and untrustworthy stimuli were systematically confounded with the presence of facial happiness and anger, respectively. These results raise the possibility that the preferential looking results described simply reflect an early preference for positive facial affect^32–34^.

It is important that future studies of the development of first impressions distinguish trait inferences based on facial structure from those based on facial expression^26^. These two types of trait inference are likely to be mediated by different neurocognitive mechanisms and may exhibit different developmental trajectories. Where the interpretation of empirical findings rests on the facial stimuli being emotion neutral–or perhaps more likely, that expression cues do not vary systematically between conditions–it is imperative that authors evidence this key claim using rigorous and sensitive procedures.

## Data Availability

The data underlying the analyses described can be accessed via the Open Science Framework (https://osf.io/dpxgw/?view_only=f2b94b1d60994f568d521632fc151868).
